# Highly efficient passive Tesla valves for microfluidic applications

**DOI:** 10.1038/s41378-022-00437-4

**Published:** 2022-09-07

**Authors:** Sebastian Bohm, Hai Binh Phi, Ayaka Moriyama, Erich Runge, Steffen Strehle, Jörg König, Christian Cierpka, Lars Dittrich

**Affiliations:** 1 Theoretical Physics I, Technische Universität Ilmenau, Weimarer Straße 25, 98693 Ilmenau, Germany; 2 Research and Development, 5microns GmbH, Margarethenstraße 6, 98693 Ilmenau, Germany; 3 Institute of Micro- and Nanotechnologies, Gustav-Kirchhoff-Straße 7, 98693 Ilmenau, Germany; 4 Microsystems Engineering, Technische Universität Ilmenau, Max-Planck-Ring 12, 98693 Ilmenau, Germany; 5 Physics and Astronomy, Carleton College, One North College, Northfield, 55057 Minnesota USA; 6 Engineering Thermodynamics, Technische Universität Ilmenau, Am Helmholtzring 1, 98693 Ilmenau, Germany

**Keywords:** Engineering, Microfluidics, Nanofluidics

## Abstract

A multistage optimization method is developed yielding Tesla valves that are efficient even at low flow rates, characteristic, e.g., for almost all microfluidic systems, where passive valves have intrinsic advantages over active ones. We report on optimized structures that show a diodicity of up to 1.8 already at flow rates of 20 μl s^−^^1^ corresponding to a Reynolds number of 36. Centerpiece of the design is a topological optimization based on the finite element method. It is set-up to yield easy-to-fabricate valve structures with a small footprint that can be directly used in microfluidic systems. Our numerical two-dimensional optimization takes into account the finite height of the channel approximately by means of a so-called shallow-channel approximation. Based on the three-dimensionally extruded optimized designs, various test structures were fabricated using standard, widely available microsystem manufacturing techniques. The manufacturing process is described in detail since it can be used for the production of similar cost-effective microfluidic systems. For the experimentally fabricated chips, the efficiency of the different valve designs, i.e., the diodicity defined as the ratio of the measured pressure drops in backward and forward flow directions, respectively, is measured and compared to theoretical predictions obtained from full 3D calculations of the Tesla valves. Good agreement is found. In addition to the direct measurement of the diodicities, the flow profiles in the fabricated test structures are determined using a two-dimensional microscopic particle image velocimetry (μPIV) method. Again, a reasonable good agreement of the measured flow profiles with simulated predictions is observed.

## Introduction

Passive valves without any moving components are an intriguing technological and economical alternative to active valves. Such valves are also called Tesla valves in honor of their inventor Nikola Tesla^1^ and are still the subject of current research today^2–4^. Two obvious advantages are the high robustness and the simple manufacturing. Even more important for applications in micro-, nano-, or even pico-fluidics is that it is intrinsically much more difficult to miniaturize valves with active components than passive designs. Unfortunately, established designs for passive valves can not simply be scaled down because they tend to loose their functionality on smaller scales with naturally smaller Reynolds numbers^5^. Miniaturization of passive valves, thus, calls for new valve designs.

The rectifying effect of Tesla valves is not based on any moving components, but relies solely on inertial forces and the resulting viscous losses, which are asymmetric for the two flow directions. This asymmetry leads to the effect that the fluid can flow rather easily through the valve in one direction, namely the forward direction, whereas the fluid experiences a significantly greater flow resistance in the reverse direction, hereinafter referred to as the backward direction. The ratio of these flow resistances describes the efficiency of a Tesla valve and is called the diodicity *Di*.

The development of efficient valves is an area of active research with many recent publications^5–9^. For practical applications, diodicities of order 2 have been reported for the Reynolds number range of *Re* ≈ 100. This work focuses on flow rates smaller than 20 μl s^−1^, which correspond to a Reynolds number of less than or about 35, with the exact value depending on the choice of the characteristic length and velocity. This parameter range presents a particular challenge for the design of efficient passive microvalves: While for *Re* ≳ 100, various simulations provide sufficiently large *Di* ≳ 2^7–9^, simulations for *Re* ≈ 50 consistently find only marginal diodicities *Di* ≳ 1.0^5,6,10^. A paradigmatic application of Tesla valves is the rectification of the volume flow in, e.g., membrane-driven^11,12^ or piston-driven micropumps^13^. The idea of passive rectification can be taken a step further by combining Tesla valves with a non-mechanical pumping principle^14,15^. For the concept of such an electrokinetic pump, this type of valve is an excellent candidate, as the same manufacturing processes are used for the fabrication of the pump and the valves. While the present manuscript focuses on Tesla valves for micropumps with flows at small Reynolds numbers (*Re* < 35), our design strategies and objective functions can also be applied and adapted to other passive non-mechanical microfluidic devices such as micromixers^16–19^ or gas decompression, e.g. in hydrogen fuel cells^20^. All these and similar applications will profit from the increased robustness and reliability, as well as the smaller footprint and inexpensive manufacturing resulting for non-mechanical static designs.

Fundamental research on the working principle of the valves was done by Bardell^21^. Gamboa et al.^17^ and Truong et al.^22^ worked on the optimization of Tesla valves starting from the original design of Nikola Tesla. Lin et al.^23^ proposed a topological optimization method to increase the diodicity even further. Their valve designs achieve very high diodicities at medium to high flow rates. A remaining major challenge lies in the fact that the achievable diodicity of Tesla valves depends strongly on the flow rates and is usually very low for small flow rates. Tesla diodes can work in practice only when the Reynolds number is larger than one, i.e. inertia plays a role. If the Reynolds number becomes close to unity, the flow is typically reversible and the Tesla diode shows the same pressure drop in both directions^24^. In a simplified geometry (e.g. nozzle-diffusor valves) the diodicity is expected to increase linearly with Reynolds number. However, for more complex structures optimal Reynolds number ranges can be determined. Typically the mass-flow rate or volume flow rate is controlled in microfluidics. Both correspond to each other for liquids as in typical pressure ranges for microfluidics the liquids can be considered to be incompressible. Assuming a constant volume flow rate, channel height, and fluid properties, the Reynolds number is inversely dependent on the channel width. In Tesla valves the channel width changes with streamwise direction. Therefore, the Reynolds number should be determined at the minimum cross-section or the inlet of the Tesla diode. We decided to use the inlet as reference as also done in the literature^10,23^ to be able to compare the different geometries here. Unfortunately, in microfluidic systems flow velocities are typically small, making the use of Tesla valves inefficient up to now. This problem was already recognized by Deng et al.^25^. They used a topological optimization method for the design of Tesla valves, which should be efficient for small Reynolds numbers. However, the achievable diodicities are not particularly high (of order ≈ 1.25) even with a twelve-stage valve design. Furthermore, the early research indicated the necessity of a relatively large size for efficient valves, which counteracts the purpose of microfluidics. Another approach was presented by Tao et al.^26^, who used asymmetric converging-diverging microchannels to fabricate efficient valves for small flow rates. This approach already yields high diodicities of maximum 1.77 at moderate flow rates but is still not efficient enough at very low flow rates. A comprehensive study of existing passive valves at low Reynolds numbers was performed by Fadl et al.^27^, and none of the structures studied showed a sufficiently high diodicity. In this paper, an improved multi-stage optimization procedure is presented, which generates Tesla valves that are efficient even at very low flow rates or Reynolds numbers. At the same time, these valves have a small footprint and are easy to manufacture. Different valve designs are shown and characterized by fluidic measurements. Besides the direct measurement of the diodicities, the flow profiles are determined by micro-particle image velocimetry (μPIV) measurements and compared with the simulated predictions. We start with a description of the topological optimization. While the optimization is done quasi-two-dimensional, taking into account the finite height only approximately, the subsequent simulations predicting the properties of the optimized designs are done fully three-dimensionally. Thereafter, we present a discussion and experimental verifications for both diodicities and velocity profiles. Finally, we summarize the materials and methods used.

## Results

### Topological optimization

**Fig. 1:**
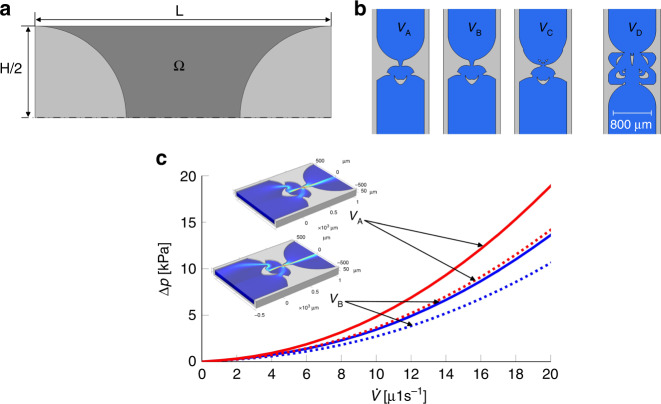
Simulation domain and examples for optimized valve structures. **a** Schematic representation of the simulation domain with the length *L* and the height *H*. A symmetrical valve design is aimed at and thus the simulation on one half of the geometry is sufficient. The dark grey area shows the design domain used for the topological optimization. **b** Examples of the optimized valve geometries *V*_A_, *V*_B_, *V*_C_ and the geometry *V*_D_. Geometry *V*_D_ is based on the optimization algorithm introduced by Lin et al.^23^ and is efficient at high Reynolds numbers or flow rates. It serves as a benchmark for comparisons. The differences between valve geometry *V*_A_ and *V*_B_ are only marginal and difficult to see with the naked eye. However, even these small differences lead to a strong decrease in the fluidic resistance for valve design *V*_B_. This underlines the fact already found by Lin et al. that the efficiency of the Tesla valves is very sensitive to even slight changes in the geometry. This highlights the importance of precise manufacturing of the Tesla valves. **c** Simulated pressure differences as a function of the flow rates for different flow directions for the optimized valve designes *V*_A_ and *V*_B_. Solid lines show the pressure drop for the forward direction and dashed lines show the pressure drop for the backward direction. For the optimization of valve design *V*_B_, the pressure difference in the forward direction was minimised during the final optimization by setting *w*_4_ = 1 in Eq. (9). In contrast, the factor *w*_4_ was set to zero for the optimization of valve *V*_A_. It becomes clear that the fluidic resistance of valve *V*_B_ is significantly lower. At the same time, the diodicity is only slightly lower, cf. Fig. 3a below.

This section explains the proposed algorithm for the optimization of Tesla valves. Topological optimization^28^ methods have already been proven to be useful for dimensioning efficient passive valves. A seminal paper on this subject was written by Borrvall et al.^29^. The method was subsequently refined by Lin et al.^23^. However, the method described therein does not provide efficient valve designs at small flow rates. This can be explained by the fact that for small flow rates, the pressure difference between the forward and backward directions becomes smaller, making the optimization problem more difficult. To address these issues, a multi-stage optimization procedure with a customized objective function was developed. In addition, we found the choice of the form of the optimization region to play a decisive role for the result. A typical variant for the possible choice of the optimization area which leads to small and efficient valve designs at the same time is presented in Fig. 1a. For the optimization, it is necessary to define what an ’optimal valve design’ actually means. In the past, the requirement was primarily to achieve a high diodicity, defined as the ratio of the pressure differences between the forward and backward directions at a constant flow rate:

1 $${\left.Di = \frac{{{\Delta }}{p}_{{{{\rm{B}}}}}}{{{\Delta }}{p}_{{{{\rm{F}}}}}}\right|}_{\dot{V} = {{{\rm{const.}}}}}$$

However, valve designs that show only high diodicity are usually not applicable in a realistic microfluidic system. In addition to high diodicity, usually a low overall fluidic resistance in the forward direction is required. Further conditions such as the minimum structure size matter for manufacturing. For actual applications, the filling of the system with a liquid should be possible without forming any air bubbles inside the structures. All these and similar requirements can be accounted for by a target-oriented choice of the objective function combined with a multi-stage optimization procedure.

For the optimization, a region is first defined where the geometry can be changed during the optimization. This area is called the design domain. Having typical micro-fluidic systems in mind and in view of the numerical effort, we limit ourselves here to two-dimensional design domains. In the design domain, a function *γ*_C_ is introduced which describes the material distribution: A value of *γ*_C_ = 0 shows that there is material in the respective point and a value of *γ*_C_ = 1 means that the fluid can flow freely. In order to be able to apply a derivative-based optimization procedure, the permissible range of *γ*_C_ is extended to the whole range 0 ≤ *γ*_C_ ≤ 1. Nevertheless, the goal of the final design is for the function *γ*_C_ to converge to either 0 or 1 at each point. The optimization task is now to find the optimal value of the *γ*_C_ on each mesh element within the design domain. However, the direct use of this approach would lead to the material distribution being strongly dependent on the size of the mesh elements used. In addition, very small and highly porous structures would possibly be obtained, which cannot be manufactured. Therefore, the material distribution *γ*_C_ is filtered and, thus, smoothened by solving the Helmholtz PDE^30–32^

2 $${\gamma }_{{{{\rm{F}}}}}={\gamma }_{{{{\rm{C}}}}}+{R}_{\min }^{2}{\nabla }^{2}{\gamma }_{{{{\rm{F}}}}}$$

where *γ*_F_ is the filtered material distribution and ${R}_{\min }$ is a minimum length scale. Good results are obtained if the filter length is chosen equal to the minimum mesh length *m*_sz_. However, this filtering leads to the fact that *γ*_F_ can take values greater than 1 or less than 0. For this reason, the function is projected back to the range from 0 to 1 using a hyperbolic tangent function by solving the equation

3 $${\gamma }_{{{{\rm{P}}}}}=\frac{\tanh (\beta ({\gamma }_{{{{\rm{F}}}}}-{\gamma }_{{{{\rm{\beta }}}}}))+\tanh (\beta {\gamma }_{{{{\rm{\beta }}}}})}{\tanh (\beta (1-{\gamma }_{{{{\rm{\beta }}}}}))+\tanh (\beta {\gamma }_{{{{\rm{\beta }}}}})}$$

where *γ*_P_ is the back-projected material distribution, *γ*_β_ is the projection point and β is the projection slope that controls the projection steepness.

The profile *γ*_P_ is now used as input for the fluid-dynamic calculations. To make the flow dependent on the material distribution, a Darcy force ${F}_{{{{\rm{D}}}}}={\sum }_{i}\alpha ({\gamma }_{{{{\rm{P}}}}}){u}_{i}^{2}$ ^33,34^ is introduced, where *α* depends nonlinearly on the projected material distribution *γ*_P_ as follows:

4 $$\alpha ({\gamma }_{{{{\rm{P}}}}})={\alpha }_{\max }q\frac{1-{\gamma }_{{{{\rm{P}}}}}}{q+{\gamma }_{{{{\rm{P}}}}}}$$

The quantity *q* is a freely chooseable factor that controls the convexity of the function, which must, however, be chosen appropriately in order to obtain meaningful designs. The factor ${\alpha }_{\max }$ controls how much the liquid can penetrate into the solid region. A very high value for ${\alpha }_{\max }$ is desirable to imitate the behavior of an impenetrable solid material, however a too large value of ${\alpha }_{\max }$ causes the solver to converge to a local minimum early. In the method presented here, the value of ${\alpha }_{\max }$ is adjusted during the optimization process. To be specific, ${\alpha }_{\max }$ is chosen dependent on the size of the mesh elements as

5 $${\alpha }_{\max }={\alpha }_{0}\frac{\eta }{{m}_{{{{\rm{sz}}}}}^{2}}$$

where *η* is the viscosity of the liquid and *α*_0_ is a freely chooseable parameter. Borrvall and Petersson^29^ have shown that direct maximization of the diodicity according to Eq. (1) constitutes an ill-posed optimization problem. Instead, they proposed to optimize the ratio of the energy dissipations for the two flow directions

6 $${{\Delta }}{E}_{{{{\rm{Disp}}}}}[{\gamma }_{{{{\rm{P}}}}}]=\frac{\int \frac{\eta }{2}{\left(\frac{\partial {u}_{i,{{{\rm{B}}}}}}{\partial {x}_{k}}+\frac{\partial {u}_{k,{{{\rm{B}}}}}}{\partial {x}_{i}}\right)}^{2}+\mathop{\sum}\limits_{i}\alpha ({\gamma }_{{{{\rm{P}}}}}){u}_{i,{{{\rm{B}}}}}^{2}{{{\rm{d}}}}V}{\int \frac{\eta }{2}{\left(\frac{\partial {u}_{i,{{{\rm{F}}}}}}{\partial {x}_{k}}+\frac{\partial {u}_{k,{{{\rm{F}}}}}}{\partial {x}_{i}}\right)}^{2}+\mathop{\sum}\limits_{i}\alpha ({\gamma }_{{{{\rm{P}}}}}){u}_{i,{{{\rm{F}}}}}^{2}{{{\rm{d}}}}V}$$

where the Einstein summation convention is used, *u*_*i*_ are the components of the velocity field, and the indices B and F refer to the backward and the forward direction, respectively. Lin et al.^23^ showed under which assumptions both objective functions lead to the same result. The Borrvall-Petersson approach is also used here, but the objective function is extended by further terms. The flow profiles entering, e.g., Eq. (6) are obtained by solving the laminar Navier-Stokes equation

7 $$\varrho(\overrightarrow{u}\cdot \nabla )\overrightarrow{u}=-\nabla p+\eta {\nabla }^{2}\overrightarrow{u}-\alpha ({\gamma }_{{{{\rm{P}}}}})\overrightarrow{u}-12\frac{\eta }{{d}_{z}^{2}}\overrightarrow{u}$$

twice in each optimization iteration. The term $-\alpha ({\gamma }_{{{{\rm{P}}}}})\overrightarrow{u}$ corresponds to the Darcy force, which couples the material distribution to the flow profile. The last term $-12(\eta /{d}_{z}^{2})\,\overrightarrow{u}$ results from a so-called shallow-channel approximation^34^. This term is introduced in order to at least partially take into account the influence of the low channel height during the two-dimensional simulations used for the optimization. For small flow rates, our experience shows that the direct use of Eq. (6) as an objective function is not effective. Therefore, the following adjusted objective function is used:

8 $$Z[{\gamma }_{{{{\rm{P}}}}}]={w}_{1}{{\Delta }}{E}_{{{{\rm{Disp}}}}}(\alpha ({\gamma }_{{{{\rm{P}}}}}))+{w}_{2}\frac{{{\Delta }}{p}_{{{{\rm{B}}}}}}{{{\Delta }}{p}_{{{{\rm{F}}}}}}+\frac{{w}_{3}}{{A}_{{{{\rm{O}}}}}}\int \left(1-{\gamma }_{{{{\rm{P}}}}}\right){{{\rm{d}}}}A$$

This objective function now consists of three different terms. The parameters *w*_1,2,3_ ∈ [0, 1] are weighting factors for the different, partly competing design goals. The first term corresponds to the ratio of the energy dissipation according to Eq. (6). The second term corresponds to the actual optimization of the diodicity according to Eq. (1). Since the optimization of energy dissipation does not exactly correspond to the optimization of diodicity, this term is helpful to guide the optimizer in the right direction. The third term favours designs that fill as much of the structure as possible with material in order to ease fabrication: *A*_O_ is the total area of the design domain. Together with the filtering of the material distribution via Eq. (3), this term prevents structures that are too small for actual fabrication.

Using this objective function, a multi-stage optimization procedure is carried out, which adjusts the value of the minimum mesh length *m*_sz_ in each step. First, the value of *m*_sz_ is chosen rather large and then gradually reduced. This leads to the effect that there are fewer mesh elements at the beginning and the search space is thus comparatively small. In addition, the liquid can according to Eq. (5) penetrate the material stronger, which increases the robustness of the optimization at the beginning. In each subsequent optimization step, the previously found material distribution is used as a starting point for further optimization and the mesh size is reduced. A possible problem with this method is that only a single flow rate ${\dot{V}}_{0}$ is taken into account during optimization. Thus, a single operating point of the valve must already be determined at the beginning. This leads to the fact that the diodicity can be very high for the specific flow rate but at the same time, the response curve is usually steep and drops off quickly for smaller flow rates. However, in many cases, valve designs that work efficiently over a wide range of flow rates are desirable. In addition, a strongly non-linear response curve leads to a strongly non-linear system behavior, which is often not wanted. For these reasons, a further optimization step with an adjusted objective function is carried out within our multi-stage optimization. This last stage uses the following objective function where the optimization is now carried out over a given range of flow rates simultaneously:

9 $$Z[{\gamma }_{{{{\rm{P}}}}}]=\mathop{\sum }\limits_{i=1}^{N}\left[\frac{{\dot{V}}_{\max }}{{\dot{V}}_{i}}\frac{{{\Delta }}{p}_{i,{{{\rm{B}}}}}}{{{\Delta }}{p}_{i,{{{\rm{F}}}}}}+{w}_{4}\log {\left(\frac{{{\Delta }}{p}_{i,{{{\rm{F}}}}}}{\varrho {u}_{{{{\rm{in}}}},i}^{2}}\right)}^{-1}\right]$$

Here, the set of flow rates ${\dot{V}}_{i}$, *i* = 1, …*N* is specified in a given range of interest. Since an efficient valve design is already available at this point of the multistage optimization, the diodicity instead of the ratio of the energy dissipation can now be maximized directly, which is expressed by the first term. In order to increase the influence of the usually small diodicities at small flow rates to the objective, the maximum flow rate ${\dot{V}}_{\max }$ divided by the *i*-th flow rate ${\dot{V}}_{i}$ is used as a weighting factor for the diodicity. In addition, another term is introduced which favours the minimization of the pressure difference in the forward direction. In contrast to solely optimizing the diodicity, not only the ratio of the pressure differences but also a small total fluidic resistance plays a relevant role. Deng et al.^25^ showed the usefulness of this additional optimization term. To weight these terms similarly, the pressure difference is divided by the inlet velocity *u*_in_ for the *i*-th flow rate multiplied by the density of the liquid *ϱ*. In addition to the choice of objective functions, the form of the design domain plays a decisive role for the result. The form of the design domain used here is shown in Fig. 1a. This curved shape of the design domain was chosen because the normally used sharp-edged design domains, see e.g. Lin et al.^23^, lead to air inclusions and bubbles during the filling process of actual manufactured structures. In summary, this study presents the following four novelties in order to find designs of efficient microfluidic Tesla valves at low flow rates that are still easy to manufacture:

1. A customized objective function that simultaneously maximizes the diodicity and energy dissipation and also prevents the formation of small structures.
2. A multi-stage optimization procedure in which the element size of the underlying mesh is reduced successively.
3. A final optimization step in which the diodicity is simultaneously maximized over a certain flow rate range and, if desired, the fluidic resistance is also minimized in the direction of flow.
4. An appropriate choice of the shape of the design domain, which favors designs that allow easy manufacturing and a bubble-free filling process.

### Simulation results

**Fig. 2:**
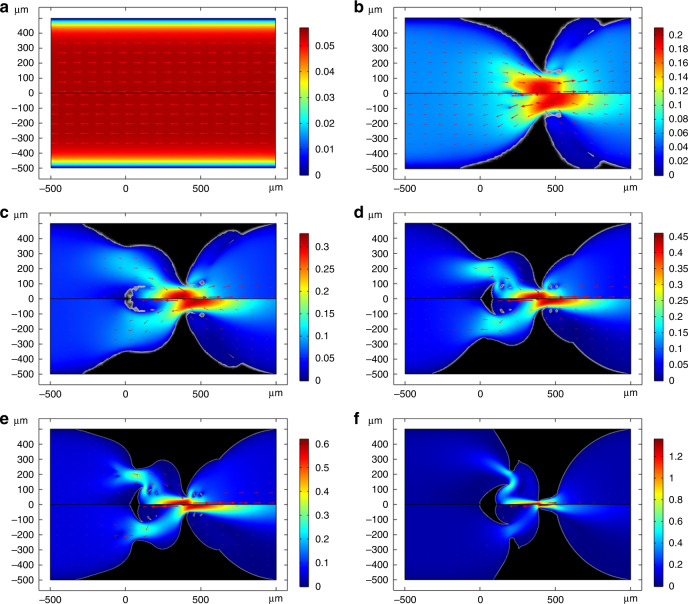
Images of the calculated material distribution together with the flow profiles during the individual stages of the optimization process. In each image, the upper half shows the flow in the backward direction and the lower half the flow in the forward direction. The color shows the norm of the velocity field and the arrows indicate the velocity vectors. The initial flow profile is shown in **a**. Figures **b**–**e** shows the results of the optimization at the end of each optimization stage using Eq. (8). Figure **f** shows the result after the final optimization step using the objective function of Eq. (9). A three-dimensional model can then be obtained from such a design by extrusion, whereas the two-dimensional geometry is sufficient for manufacturing by means of lithographic processes.

In this section, exemplary simulation results and the parameters used are presented. All simulations were carried out using the finite element software COMSOL Multiphysics^®^ (the Supplementary Information provides more details about the implementation in COMSOL). For the optimization, the gradient-based Sparse Nonlinear OPTimizer SNOPT^35^ is used. The meshing is done with triangular elements. Constant shape functions are used to approximate the material distribution function *γ*_P_ and first order elements are used to discretize the velocity and the pressure field. At the beginning, the material distribution on the entire design domain is initialized with a value of 1, which means that there is no material within the design domain at the beginning. A projection slope *β* = 8 and the projection point *γ*_β_ = 0.5 are used. A value of *q* = 1 is set for the Darcy penalization. In the first step, a certain number of optimization steps are carried out while using a single fixed flow rate value. Each optimization step consists of a given number of optimization iterations. Our experience suggests that a four-stage optimization procedure is a good trade-off between computational effort and valve efficiency. Between two successive optimization steps, the size of the mesh elements is reduced step-wise by 5 μm from an initial value of 20 μm to 5 μm. A maximum of 500 iterations are performed in the first four optimization stages. Following the four-stage optimization using the objective function defined by Eq. (8), a further optimization step is carried out using Eq. (9) as objective function. A maximum of 1000 iterations are performed in this last optimization step. For all simulations presented in this manuscript, *α*_0_ was set equal to 25. Trying different values, we found that good results are obtained for *w*_1_ = *w*_2_ = 1 and *w*_3_ = 0.05. Examples of the simulation results after each step of the optimization are shown in Fig. 2a–f (as Supplementary Information a video is available, which shows the material distribution during the optimization). It can be seen that a diode-like structure is formed at an early stage. In the progress of the optimization, additional island structures already known from conventional topological optimizations are added to the structure. One notices that many small additional structures are formed by the software before the final optimization step. However, these are removed again automatically in the last optimization step, as they do not improve the diodicity at small flow rates. It becomes clear that the optimization process favors nozzle-like structures. These nozzles cause the flow velocity to be greatly increased locally. Together with the ‘cleverly’ placed obstacles, this greatly increases the flow resistance in the backward direction.

Three different valve structures for different parameter combinations are shown in Fig. 1b. The parameters used for the optimization are summarized in Table 1. For the optimization of design *V*_A_ and *V*_B_, all weights except *w*_4_ were chosen identically. The resulting performance difference highlights the influence of the additional minimization of the pressure difference in the forward flow direction in the final optimization step: Both designs show only minimal geometric differences leading to an also almost identical diodicity. However, the pressure difference for the two flow directions is significantly lower for design *V*_B_ as shown in Fig. 1c.

**Table 1:** Parameters used for the optimization of the valve designs *V*_A,B,C_

Parameter	Valve designs		
	*V*_A_	*V*_B_	*V*_C_
*w*_1_	1	1	1
*w*_2_	1	1	1
*w*_3_	0.05	0.05	0.05
*w*_4_	0	1	0
${\dot{V}}_{0}$ [μL s^−1^]	7.5	7.5	7.5
${\dot{V}}_{i}$ [μL s^−1^]	[1,...,7.5]	[1,...,7.5]	[2.5,...,10]
*N*	10	10	5

The described optimization algorithm yields two-dimensional geometries at first. Real manufactured valve structures, on the other hand, are always three-dimensional and, in the case of microfluidic systems, the aspect ratio of channel width to channel height is usually very high. Even though the additional term $-12\frac{\eta }{{d}_{z}^{2}}\overrightarrow{u}$ in Eq. (7) already partially accounts for the finite channel height during optimization, an exact agreement in the three-dimensional case with the optimization results cannot be expected. In order to simulate the diodicity as accurately as possible, the two-dimensional material distributions were translated into three-dimensional geometries. For this purpose, the isoline *γ*_P_ = *γ*_th_ is determined where *γ*_th_ is a threshold value. The resulting curve provides closed areas which are then extruded and subtracted from the extruded initial simulation area. In all results shown below, a value of *γ*_th_ = 0.5 was chosen. The threshold value plays only a minor role for the final geometry, since the presented optimization algorithm provides very sharp transitions in the material distribution. Finally, for the three-dimensional valve geometries, the flow rate-dependent diodicity can be calculated numerically: For this purpose, the Navier-Stokes equations $\varrho (\overrightarrow{u}\cdot \nabla )\overrightarrow{u}=-\nabla p+\eta {\nabla }^{2}\overrightarrow{u}$ are solved for both flow directions and the pressure differences are determined. All the results shown subsequently, as well as the pressure curves in Fig. 1c, were determined for the three-dimensional geometries. To validate the simulation results, the dependence of the shape of the isolines on the choice of the threshold value *γ*_th_ and the dependence of the results of the three-dimensional simulations on the mesh size were investigated. The results of the convergence study are summarized in the Supplementary Information.

## Discussion

### Pressure measurements and diodicity

In this section, measurement results gained from fabricated test chips are compared with simulation results. The diodicities were determined by measuring the pressure drops for the both flow directions as described in the methods section of the diodicity measurement. An example for the pressure drops for a range of flow rates is show in Fig. 5b. As expected, the flow resistance increases with an increasing flow rate. The typical non-linear behavior of the Tesla valves is apparent. In addition, it can be seen that the backward direction shows a significantly higher flow resistance. The measured pressure curves can be used to directly calculate the diodicity. However, since the pressure was not measured directly at the outlet or inlet of the valves, there were additional pressure drops Δ*p*_ind_ independent of the flow direction. In this case, the measurable diodicity is given by:

10 $$D{i}_{{{{\rm{meas}}}}}=\left(\frac{{{\Delta}}{p}_{{{{\rm{B}}}}}+{{\Delta}}{p}_{{{{\rm{ind}}}}}}{{{\Delta }}{p}_{{{{\rm{F}}}}}+{{\Delta}}{p}_{{{{\rm{ind}}}}}}\right)\Big| {}_{\dot{V} = {{{\rm{const.}}}}}$$

**Fig. 3:**
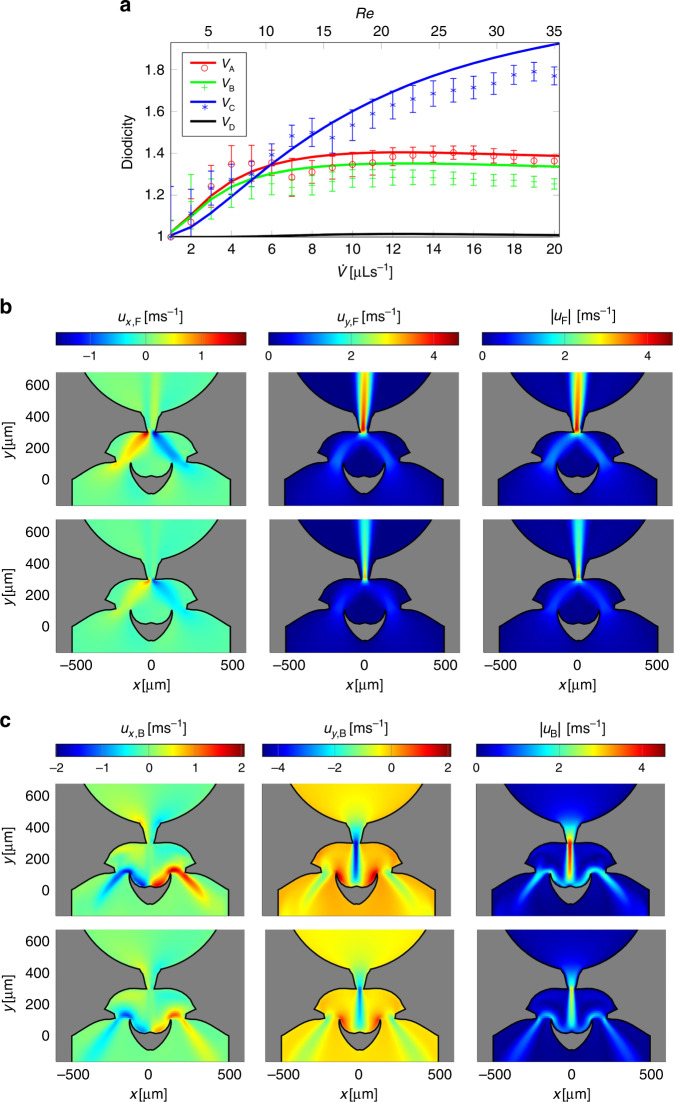
Comparison between simulated and measured results. **a** Comparison between the measured and simulated diodicities as a function of flow rate as well as the Reynolds number for the different valve designs. Solid lines show the simulation results and individual points show the respective measured values. The plotted points are averaged values of three measurement series for each flow rate for each design. In addition, the standard errors for each measuring point are shown. As expected, the measurement error is larger for small pressure values. The maximum flow rate corresponds to a Reynolds number *Re* = *ϱ* ⋅ *U* ⋅ *d*_H_ / *η* of approximately 36 where *d*_H_ = 2*h*_C_*l*_C_ / (*h*_C_ + *l*_C_) is the hydraulic diameter. The Reynolds number is calculated at the inlet of the valve with a length *l*_C_ = 1000 μm and a height of *h*_C_=130 μm (a more detailed discussion can be found in the Supplementary Information). **b**,**c** Comparison between the flow profiles measured by μPIV and the simulated flow profiles for the valve *V*_A_ (the Supplementary Information shows the measurement results for the valve designs *V*_B_ and *V*_C_). In the upper rows of the two subfigures, the measured data are shown and in the bottom rows, the simulation results are presented. In all illustrations, the valve geometries were overlaid to facilitate visualization. The individual velocity components *u*_*x*,*y*_ and the magnitude ∣*u*∣ of the velocity vectors are displayed. **b**,**c** shows the flow profiles for the forward and the backward direction, respectively.

In the simulations, the contribution Δ*p*_ind_ is zero by definition and Eq. (10) reduces to Eq. (1). Since the term Δ*p*_ind_ in the measurement is small but not zero, an exact agreement with the simulation results cannot be expected, and indeed the measured diodicity turned out to be smaller than the simulated diodicity. Figure 3a shows the comparison between the measured and simulated diodicities for the different valve designs. For a given geometry the Reynolds number depends only on the volume flow rate and the upper *x*-axis presents the inlet Reynolds number. Even though the measured diodicities are, as discussed, usually slightly smaller, there is very convincing agreement between the simulation and the measurement results. It can be seen that a minimal diodicity of 1.3 can be reached for all geometries for inlet Reynolds numbers between 10 and 35 for three different valve designs *V*_A_, *V*_B_, and *V*_C_. Valve designs *V*_A_ and *V*_B_ show a rapid increase in diodicity for small flow rates, which is subsequently followed by a plateau of the diodicity. In contrast, the diodicity for design *V*_C_ increases slower with Reynolds number (or volume flow rate) but finally reaches significantly larger values. This can be explained by the fact that the final optimization step used a larger flow rate range (see Table 1). The results show that a measured diodicity of approximately 1.8 is achieved at a flow rate of only 20 μl s^−1^ (corresponding to a Reynolds number of approximately 36 calculated at the inlet), which to our knowledge is currently the highest diodicity at such low flow rates and Δ*Di* = *Di* − 1 is between two to three times as high as the diodicities reported before. At the same time, the absolute pressure drop is low, making the valves ideal for microfluidic applications.

### Velocity field measurements

In addition to the measurement of the pressure differences for the determination of the diodicities, the velocity fields were measured using two-dimensional μPIV with the setup described in the methods section of the velocity measurement. The measured velocity fields can be directly compared with the simulated velocity fields and they show a good qualitative agreement. In Fig. 2b, c, the measured velocity is shown for design *V*_A_ in the upper row of the subfigures for forward (b) and backward (c) flow. The data for the other designs are presented in the Supplementary Information. What becomes obvious already from the visual inspection is that the magnitude of the velocity in the experiment is always a bit higher than in the simulation. The mean ratio between the measured and simulated velocities is 1.45. The higher velocity can be explained due to the actual channels being smaller in height compared to the numerical simulation. The smaller height is caused by the etching and the processing of the Ordyl bond medium that typically shrinks during the bonding or becomes thinner during the development process. As the volume flow rate was controlled in the experiment a smaller cross-section results in larger velocities. To quantitatively assess the agreement in the patterns a correlation between the experimental and simulated velocity fields was performed (for more details see Sachs et al.^36^). This analysis gives correlation coefficients^37^ of ≥95% for the data of Fig. 3 for both directions, where a correlation of 100% would stand for a perfect agreement. As the flow topology in the investigated Reynolds number range does not change significantly the μPIV-experiments validate the numerical simulations and the whole optimization approach can be expected to result in optimized geometries even if small tolerances in the final velocity field due to the fabrication arise.

In the paper, a robust topological optimization procedure was introduced for designing Tesla valves which are efficient even at low flow rates. To the best of our knowledge, the proposed process provides the most efficient valve designs currently available in this flow-rate range. The resulting designs show only large free-standing island structures and are thus comparatively easy to manufacture and simultaneously offer a small footprint. Thus, they are promising candidates for passive valve designs in microfluidic applications with low flow rates. The measurements of the diodicities, as well as the flow profiles on three-dimensional test structures, show good agreement with the simulation results, which confirms the predictive accuracy of the simulation. Since objective functions with different terms were used for the optimization, the final design can be influenced by the appropriate choice of weighting parameters. It should be noted explicitly that the presented multi-stage optimization procedure can also be applied for designing Tesla valves which are efficient at higher flow rates and at the same time simple to manufacture. We also stress again that only readily available and economically viable fabrication steps were used for the valves produced for experimental verification.

## Material and methods

### Fabrication of Tesla valves

**Fig. 4:**
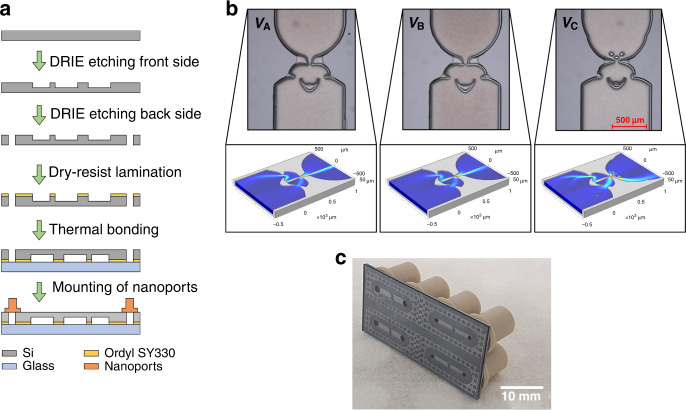
Manufacturing process and sample images of fabricated valve structures. **a** Schematic representation of the individual manufacturing steps which are carried out for the fabrication of the Tesla valves. The manufacturing process is easy to implement and consists of just a few steps. **b** Images of etched and bonded valve structures. The valves were created using the optimized DRIE etching recipe. The etched silicon structures are viewed through the glass wafer. In addition, the layer of Ordyl SY330 used for the bonding can be seen. It becomes visible that bonding does not work perfectly at the tips of the etched nozzle structures and on very small structures, as the laminate layer becomes slightly thinner during the lamination process close to the etched structures. **c** Image of a test chip with four valve structures. The nanoports, which are used for the fluidic connections, can be seen on the backside of the chip.

The Tesla valves used for the experimental characterization and verification of the optimized designs were created in a silicon substrate using a deep reactive ion (DRIE) etch process^38^. The individual steps of the manufacturing process are shown in Fig. 4a. Due to the very high aspect ratio requirement needed to create the island structures of the Tesla valves, a specially optimized etching process had to be developed. The optimization employed statistical design of experiment (DOE)^39,40^. The DRIE process is conventionally known as a two-cycle process consisting of an etch step and a passivation step, where the etch step is based on a DC Bias to physically etch the passivation layer on the bottom of the structures by accelerated ions. In contrast, the DRIE process used here consists of three steps namely, a passivation step, a breakthrough step, and a purely chemical etch step. The breakthrough step carries out the etching of the passivation layer and this step was optimized with DOE whereas standard process parameters were used for the other two steps. The etching is done at a PlasmaPro 100 Estrelas. The corresponding breakthrough parameters namely the platen power and the breakthrough time and the results are summarized in Table 2. Using the optimized etching process ‘Run 3’, island structures with lateral dimensions as small as 10 μm can be etched to a depth of 200 μm. Examples of etched Tesla valves are shown in Fig. 4b. In addition to the etching of the Tesla valves, a second DRIE process was employed to etch through holes from the backside of the substrate to create openings for the fluidic connections.

**Table 2:** Parameters used for DRIE process optimization

Run no.	RF Power (W)	Breakthrough time (s)	Sidewall angle (^∘^)
1	1500	0.5	88.9
2	1750	0.5	86.8
3	1500	0.58	87.2
4	1750	0.58	84.8
5	1625	0.54	86.3

### Wafer-bonding

The Tesla valves are sealed with a lid by bonding with a glass substrate. A transparent lid is used to allow measurements with microscopic particle image velocimetry. The island structures of the Tesla valves pose a particular challenge for the bonding. For this reason, the 30 μm-thick permanent dry film resist Ordyl SY330 was used as bonding material. In view of future applications, it should be noted that this material is biocompatible and features high chemical and thermal resistance and for these reasons has already been successfully used for the construction of microfluidic devices^41–43^. The dry film resist was laminated by a temperature of 105 °C, a pressure of approximately 0.5 bar, and a lamination speed of 1 m min^−1^ over the Tesla structures and the channels. The dry film resist that behaves like a negative resist was then structured using a conventional photolithographic process. The resist was exposed in a MA8 mask aligner from SüSS MICROTEC with a dose of 200 mJ cm^−^^2^ and developed in the Ordyl Developer XFB for a total duration of 6 min, whereby the resist only remains on the island structures of the valves and on the surfaces surrounding the fluid channels. The base and the lid wafers were then stacked together and bonded with a force of 6 kN and a temperature of 150 °C where the resist cross-links and stabilizes. To achieve complete cross-linking, the temperature of 150 °C was maintained for at least 20 min. The bonding process is visualized in the Supplementary Information. The final chip was proven to be watertight at least up to a water flow of 100 μL s^−1^, and the bonding interface was durable even against solvents such as acetone and isopropanol. After bonding was complete, the wafer stack was diced into test chips, with four valve structures on each chip. In the final step, nanoports were attached to enable a fluidic connection of the chips. An example of a finished test chip is shown in Fig. 4c.

### Diodicity measurements

**Fig. 5:**
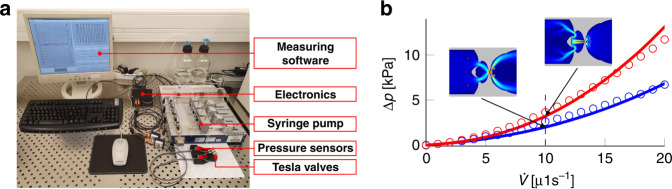
The experimental setup for the measurement of diodicity and exemplary pressure curves. **a** A syringe pump is connected to the first pressure sensor and then to the inlet of the valve. The second pressure sensor is attached to the outlet of the valve. At the end of the tubes, a small 3D-printed water sink is attached to allow the liquid to flow out without bubbles. Otherwise, due to the Laplace pressure, the bubbles would strongly influence the pressure signal. The analogue sensor signals are digitised using custom-built electronics and are automatically recorded. **b** Example of the measured and simulated pressure difference as a function of the flow rate for valve *V*_C_ for the different flow directions.

This section describes the experimental setup for the measurement of diodicity of the Tesla valves as defined by Eq. (1). Thus, for the direct measurement of the diodicity, it is necessary to measure the inlet and outlet pressure of a Tesla valve for both flow directions and calculate the ratio of the respective pressure drops. To generate a constant flow rate, a high precision neMESYS syringe pump from CETONI was used. At the same time, the pressure was measured directly before and after the fluid connections with two precise MPS-pressure sensors from Elveflow. The experimental setup is shown in Fig. 5a. The flow rate was increased by 1 μl every seven seconds from 0 μl to 20 μl. Since the pressure was initially unstable after every flow rate variation, the measured values during the first and last second of each seven-second-long measurement interval were ignored. The mean value of the pressure values was calculated from the values of the remaining five seconds of each measuring interval. The resulting mean values are shown in Fig. 5b. After measuring the pressure drop for both directions, the diodicity was calculated using Eq. (1) (see Supplementary Information for more details).

### Velocity measurements

For a detailed comparison of the theoretically designed structure and the actually obtained Tesla valves, velocity measurements were carried out using microscopic particle image velocimetry (μPIV^44–46^). For the potential of this method and recent advancements, the interested reader is referred to the results of the latest PIV challenge^47^. Small tracer particles (*d*_P_ = 1μm) doped with a fluorescent dye were suspended in de-ionized water as the working fluid. The volume flow rate was adjusted to 10 μl s^−1^ using a neMESYS syringe pump. The region of interest was observed with an inverted microscope (Zeiss Axio Observer by Zeiss GmbH) with a magnification of M = 5. A double-pulse Nd:YAG laser with wavelength of 432 nm (Innolas GmbH) was used to illuminate the particles. A dichroic mirror and a long-pass filter allow only the fluorescent light of the particles to pass to the camera sensor. Each experiment recorded 1000 double-frame images with a 12 bit Sensicam QE camera (PCO GmbH) with 1376 × 1040 pixel using a frame rate of ~ 4 Hz. The time delay between frames was in the order of 10 μs and was adjusted to the specific setup to give a mean particle image displacement of about 15 pixel. A calibration target (Thorlabs GmbH, 100 μm spacing) was used for calibration and gave a calibration factor of 1185 pixel/mm. For time synchronization and image evaluation the software DaVis (LaVision GmbH) was used. To reduce the effect of the out-of-focus particles for the cross-correlation evaluation, image pre-processing was applied (subtraction of sliding minimum for background removal, min-max intensity and bandwidth filter)^48^. As only small particles were used and the seeding concentration was kept moderate, the sum-of-correlation approach^49^ was used to give reliable results with high resolution. A multi-grid window deformation approach with decreasing interrogation window size and 50% overlap was applied to determine the large velocity range with low relative uncertainty with a final vector spacing of 6.75 μm in each direction.

## Supplementary information

Supplementary information

Video showing the optimization process

## Data Availability

The experimental data supporting the findings in this work and the custom simulation files used for the optimization are available from the corresponding author on request.

## References

[1] Tesla, N. Circuit elements having no moving parts, US Patent US1329559A (issued Feb. 3, 1920).

[2] Nguyen QM, Abouezzi J, Ristroph L (2021). Early turbulence and pulsatile flows enhance diodicity of Tesla’s macrofluidic valve. Nat. Commun..

[3] Raffel J, Ansari S, Nobes DS (2021). An experimental investigation of flow phenomena in a multistage micro-Tesla valve. J. Fluids Eng..

[4] Qian J, Chen M, Liu X, Jin Z (2019). A numerical investigation of the flow of nanofluids through a micro Tesla valve. J. Zhejiang University-Science A..

[5] Thompson M, Paudel J, Jamal T, Walters K (2014). Numerical investigation of multistaged tesla valves. J. Fluids Eng..

[6] Hu P (2022). Numerical investigation of tesla valves with a variable angle. Phys. Fluids..

[7] Zhou P, Zhang T, Simon TW, Cui T (2022). Simulation and experiments on a valveless micropump with fluidic diodes based on topology optimization. J. Microelectromechanic. Sys..

[8] Dong X, Liu X (2021). A novel check microvalve designed for non-newtonian fluids by applying an optimization algorithm. Chem. Eng. J..

[9] Gaymann A, Montomoli F, Pietropaoli M (2017). Design for additive manufacturing: Valves without moving parts. In Proc. of the ASME Turbo Expo: Turbine Technical Conference and Exposition 2017.

[10] Zhang, S., Winoto, S. H. & Low, H. T. Performance simulations of Tesla microfluidic valves. In *Proc. First International Conference on Integration and Commercialization of Micro and Nanosystems, Parts A and B*, 15–19 (ASMEDC, 2007).

[11] Stemme E, Stemme G (1993). A valveless diffuser/nozzle-based fluid pump. Sensors and Actuators A: Phys..

[12] Gerlach T (1998). Microdiffusers as dynamic passive valves for micropump applications. Sensors and Actuators A: Phys..

[13] Mohammadzadeh K, Kolahdouz EM, Shirani E, Shafii MB (2013). Numerical study on the performance of tesla type microvalve in a valveless micropump in the range of low frequencies. J Micro-Bio Robotics..

[14] Hoffmann, M., Dittrich, L. & Bertko, M. Mikropumpe zur Erzeugung einer Fluidströmung, Pumpensystem und Mikrokanalsystem, Ger. Patent DE112011104467 (issued June 1, 2017).

[15] Bohm, S., Dittrich, L. & Runge, E. Three-dimensional time resolved fluid mechanics simulation of an EWOD-driven micropump. In *Proc. COMSOL Conference 2020* (COMSOL Conference, 2020).

[16] Hong C-C, Choi J-W, Ahn CH (2004). A novel in-plane passive microfluidic mixer with modified tesla structures. Lab on a chip..

[17] Gamboa AR, Morris CJ, Forster FK (2005). Improvements in fixed-valve micropump performance through shape optimization of valves. J. Fluids Eng..

[18] Wang C-T, Chen Y-M, Hong P-A, Wang Y-T (2014). Tesla valves in micromixers. Int. J. Chem. Reactor Eng..

[19] Ameer, A. et al. Strengthening mass transfer with the tesla-valve baffles to increase the biomass yield of arthrospira platensis in a column photobioreactor. *Bioresource technol*. **320**, 124337 (2021). 10.1016/j.biortech.2020.124337 33157436

[20] Qian J-Y, Wu J-Y, Gao Z-X, Wu A, Jin Z-J (2019). Hydrogen decompression analysis by multi-stage tesla valves for hydrogen fuel cell. Int. J. Hydrogen Energy..

[21] Bardell, R. L. *The Diodicity Mechanism of Tesla-Type No-Moving-Parts Valves.* Ph.D. thesis, Univ. of Washington, Washington (2000).

[22] Truong TQ, Nguyen NT (2003). Simulation and optimization of Tesla valves. Nanotech.

[23] Lin S, Zhao L, Guest JK, Weihs TP, Liu Z (2015). Topology optimization of fixed-geometry fluid diodes. J. Mechanical Design..

[24] Troian SM (2006). Introduction to microfluidics. Phys. Today..

[25] Deng, Y., Liu, Z., Zhang, P., Wu, Y. & Korvink, J. G. Optimization of no-moving part fluidic resistance microvalves with low Reynolds number. In *Proc. 2010 IEEE 23rd International Conference on Micro Electro Mechanical Systems (MEMS),* 67–70 (IEEE, 2010).

[26] Tao R, Ng T, Su Y, Li Z (2020). A microfluidic rectifier for Newtonian fluids using asymmetric converging–diverging microchannels. Phys. Fluids..

[27] Fadl A (2009). The effect of the microfluidic diodicity on the efficiency of valve-less rectification micropumps using lattice Boltzmann method. Microsys. Technol..

[28] Chen X (2016). Topology optimization of microfluidics — a review. Microchem. J..

[29] Borrvall T, Petersson J (2003). Topology optimization of fluids in Stokes flow. Int. J. Numerical Methods Fluids..

[30] Lazarov BS, Sigmund O (2011). Filters in topology optimization based on Helmholtz-type differential equations. Int. J. Numerical Methods Eng..

[31] Wallin M, Ivarsson N, Amir O, Tortorelli D (2020). Consistent boundary conditions for pde filter regularization in topology optimization. Struct. Multidisciplinary Optim..

[32] Sigmund O, Petersson J (1998). Numerical instabilities in topology optimization: A survey on procedures dealing with checkerboards, mesh-dependencies and local minima. Structural Optimization..

[33] Whitaker S (1986). Flow in porous media i: A theoretical derivation of Darcy’s law. Transport Porous Media..

[34] Bruus, H. Theoretical microfluidics, vol. 18 of *Oxford master series in physics Condensed matter physics* (Oxford Univ. Press, Oxford, 2011), reprinted with corr edn.

[35] Gill PE, Murray W, Saunders MA (2005). SNOPT: An SQP algorithm for large-scale constrained optimization. SIAM Rev..

[36] Sachs S, Baloochi M, Cierpka C, König J (2022). On the acoustically induced fluid flow in particle separation systems employing standing surface acoustic waves - Part I. Lab Chip.

[37] Used program code can be accessed at github. https://github.com/sesa1504/AS_in_sSAW.

[38] Laermer F, Urban A (2003). Challenges, developments and applications of silicon deep reactive ion etching. Microelectronic Eng..

[39] Montgomery, D. C. Design and analysis of experiments (Wiley, Hoboken, NJ, 2013), 8 edn. http://www.loc.gov/catdir/enhancements/fy1206/2012000877-d.html.

[40] Hicks, C. R. & Turner, K. V. Fundamental concepts in the design of experiments (Oxford Univ. Press, New York, NY, 1999), 5 edn. http://www.loc.gov/catdir/enhancements/fy0602/98027756-d.html.

[41] Vulto P (2005). Microfluidic channel fabrication in dry film resist for production and prototyping of hybrid chips. Lab Chip.

[42] Vulto P, Huesgen T, Albrecht B, Urban GA (2009). A full-wafer fabrication process for glass microfluidic chips with integrated electroplated electrodes by direct bonding of dry film resist. J. Micromechanics Microeng..

[43] Günther, S. et al. EWOD system designed for optical switching. In *Proc. 2017 IEEE 30th International Conference on Micro Electro Mechanical Systems (MEMS),* 1329–1332 (IEEE, 2017).

[44] Lindken R, Rossi M, Grosse S, Westerweel J (2009). Micro-particle image velocimetry (microPIV): Recent developments, applications, and guidelines. Lab Chip..

[45] Wereley ST, Meinhart CD (2010). Recent advances in micro-particle image velocimetry. Annual Rev. Fluid Mechanics..

[46] Nabavi M (2009). Steady and unsteady flow analysis in microdiffusers and micropumps: A critical review. Microfluidics Nanofluidics..

[47] Kähler C (2016). Main results of the fourth international PIV challenge. Exp. Fluids..

[48] Rossi M, Segura R, Cierpka C, C J (2012). On the effect of particle image intensity and image preprocessing on depth of correlation in micro-PIV. Exp. Fluids..

[49] Delnoij E, Westerweel J, Deen N, Kuipers J, van Swaaij W (1999). Ensemble correlation PIV applied to bubble plumes rising in a bubble column. Chem. Eng. Sci..

