# Supplementary material for: Highly efficient passive Tesla valves for microfluidic applications
Source: Microsyst Nanoeng. 2022 Sep 7;8:97. doi: 10.1038/s41378-022-00437-4 (PMC9448783; doi:10.1038/s41378-022-00437-4)
Supplement: Supplementary file 1 — Supplementary information [file 41378_2022_437_MOESM1_ESM.pdf]

Highly efficient passive Tesla valves for microfluidic applications – Supplementary Information

Sebastian Bohm[1,2,3*], Hai Binh Phi[2,3,4], Ayaka Moriyama[5], Erich Runge[1,3], Steffen Strehle[3,4], Jörg König[3,6], Christian Cierpka[3,6] and Lars Dittrich[2]

[1]Theoretical Physics I, Technische Universität Ilmenau, Weimarer Straße 25, 98693 Ilmenau, Germany.
[2]Research and Development, 5microns GmbH, Margarethenstraße 6, 98693 Ilmenau, Germany.
[3]Institute of Micro- und Nanotechnologies, Gustav-Kirchhoff-Straße 7, 98693 Ilmenau, Germany.
[4]Microsystems Engineering, Technische Universität Ilmenau, Max-Planck-Ring 12, 98693 Ilmenau, Germany.
[5]Physics and Astronomy, Carleton College, One North College, Northfield, 55057, Minnesota, USA.
[6]Engineering Thermodynamics, Technische Universität Ilmenau, Am Helmholtzring 1, 98693 Ilmenau, Germany.

*Corresponding author(s). E-mail(s): sebastian.bohm@tu-ilmenau.de;
Contributing authors: hai-binh.phi@tu-ilmenau.de; moriyamaa@carleton.edu; erich.runge@tu-ilmenau.de; steffen.strehle@tu-ilmenau.de; joerg.koenig@tu-ilmenau.de; christian.cierpka@tu-ilmenau.de; lars.dittrich@5microns.de;

Keywords: Microfluidics, Tesla Valves, Topological Optimization, µPIV

1 Supplementary Methods

1.1 Simulation and optimization details

Some important details of the implementation of the simulation in COMSOL Multiphysics® are presented below. COMSOL specific terms and concepts are written in italics.

1.1.1 Optimization

The complete multi-stage optimization is performed using a single COMSOL file. Two *laminar fluid flow* interfaces are added to simulate the fluid flow for the forward and backward direction separately. A *fully developed flow* boundary condition is set at the inlet, where the mean flow velocity $U_{\mathrm{in}} = \dot{V}/A$ is specified. A pressure of $0\,\mathrm{Pa}$ is set at the outlet with the *suppress backflow* option selected. A *no slip* condition is set at all outer edges. To enforce symmetry, a *slip condition* is set at the center edge. The Darcy force is introduced as a volume force on the design domain. For each optimization step, a triangular mesh is introduced, where the size of the triangles on the design domain is limited by the value m_z as the maximum element size. A *study step* is introduced for each optimization step and for the calculation of the initial flow. In each *study step*, the corresponding mesh is assigned. The initial values of variables solved for are taken from the previous study step to enforce a continuous optimization. The parameters which are introduced as *local variables* and the objective functions are assigned to each *study step* by using the *modify physics* option. A *nonlocal coupling operator* is introduced to calculate the energy dissipation inside of the design domain and the mean pressure on the inlets, respectively. For the calculation of the pressures, two *average operators* are assigned to the respective boundaries of the geometry which correspond to the inlets. In order to derive a geometry from the calculated material distribution function γ_{P}, a *filter data set* with an upper bound of 0.5 is introduced in the results section. Thus, this *data set* contains a mesh representing the the solid structures. From this *data set*, a *mesh part* can be derived, which is exported as a file and serves as input for the subsequent three-dimensional fluid flow simulations.

1.1.2 Characterization of the valve structures using 3D-simulations

To create the three-dimensional COMSOL geometry, a two-dimensional *work plane* is added first. The *mesh part* from the optimization is imported onto this plane. If very small structures are present, they are removed manually. In addition, any existing interrupted line elements are repaired. Then the 2D geometry is extruded with a height of $h/2$, where h is the total height of the valve structures. The individual steps for transferring the two-dimensional geometry into a lithography mask or a 3D simulation geometry are shown in Fig. 1. Two *laminar flow interfaces* are again added to calculate the flow-rate-dependent diodicity of the three-dimensional valve structures. The *boundary conditions* are chosen corresponding to the two-dimensional simulation. To save computing time, only a quarter of the actual valve structure is simulated, with the mirror symmetry in both directions implemented by *slip conditions* on the symmetry planes. In order to determine the diodicity for different flow rates, an *auxiliary sweep* is performed.

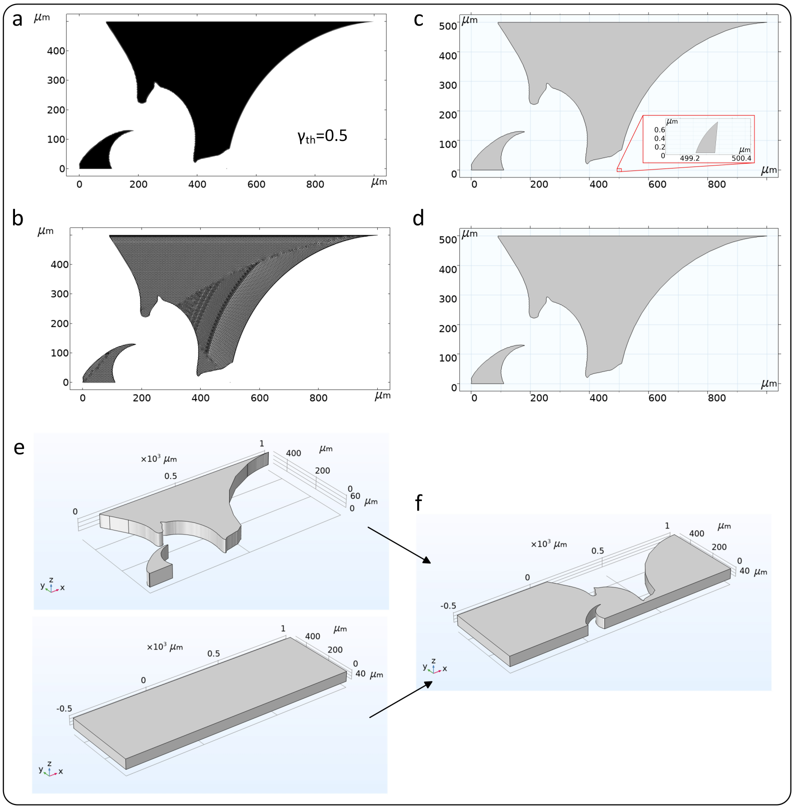

Fig. 1 Representation of the individual steps for transferring the material distribution into a mask or a 3D simulation geometry.
(a) Filtered material distribution after the completion of the topological optimization. (b) The resulting geometry from step (a) is triangulated. For mask preparation, this triangulation is translated directly into a DXF-file using an in-house developed Matlab program. To do this, the outer border of the triangulation is determined. The associated edges can be found by the fact that they are only part of a single triangle. Subsequently, the associated points are written to a DXF-file, which can be read directly by conventional CAD programs. In our case, Autocad is used for this purpose. If very small domains are present in the triangulation, they are finally removed in Autocad. (c) For the simulation of the valve performance, a three-dimensional geometry must be created. For this purpose, the mesh from step (b) is converted into a surface in COMSOL. If very small domains are present (see inset), they will be removed. The resulting geometry is shown in (d). The resulting surfaces are extruded. The three-dimensional valve structure is shown in (e). In addition, the channel is created as a 3D block. Then the valve geometry is subtracted from this block using a Boolean operation. (f) Final geometry, which is used for the simulation of valve performance. Only a quarter of the geometry is needed, since the top and the inner side represent a symmetry surface.

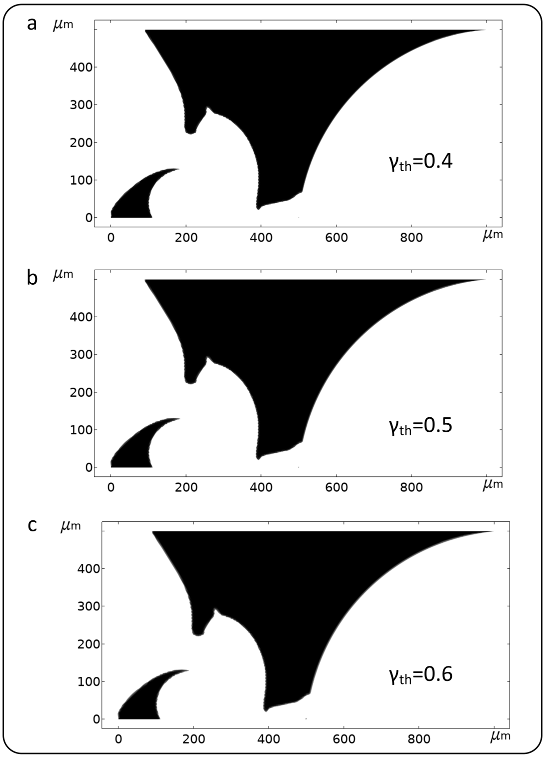

Fig. 2 Dependence of the valve geometry on the selected threshold value. The material distribution is described by the function γ_{p}. This function can take all values between 0 and 1. To derive a geometry from this, a threshold value γ_{th} must be defined. The geometry is given by the surfaces for which the condition $\gamma_{\mathrm{p}} \leq \gamma_{\mathrm{th}}$ is satisfied. Since the function is continuous, the boundary of these areas shifts slightly for a different choice of threshold value. However, the final mesh used in the topological optimization is very fine and the material distribution is well converged. Thus, the dependence of the position of the boundary on the choice of the threshold value is small and negligible compared to manufacturing tolerances. This is clearly demonstrated by the subfigures (a) to (c), where a different threshold value was used in each image: (a) $\gamma_{\mathrm{th}} = 0.4$; (b) $\gamma_{\mathrm{th}} = 0.5$; (c) $\gamma_{\mathrm{th}} = 0.6$

1.1.3 Mesh-independence study

The numerical results always show some dependence on the underlying discretization. Therefore, in order to make the results trustworthy, we performed two different convergence studies. First, we studied the geometry dependence of the two-dimensional valve geometry on the choice of the threshold value γ_{th}. Since the material distribution is given by a continuous function, the edges are not ideally sharp. However, the mesh used in the final step of the optimization is so fine that the choice of the threshold value has only a very small influence on the final geometry. This is demonstrated in Fig. 2. In addition to the choice of threshold value, we also investigated the influence of the mesh size on the 3D simulations. Since the results of the 3D simulations are used for the final comparison between experiment and simulation, a good convergence

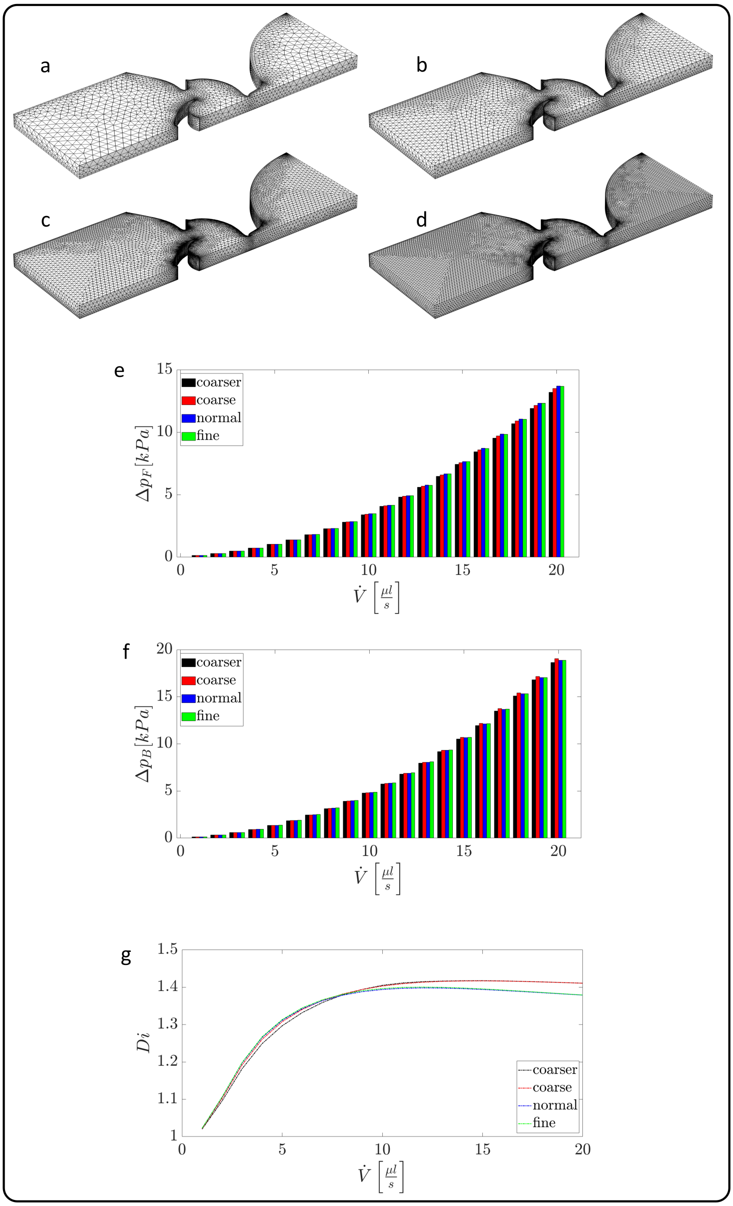

Fig. 3 Mesh independence study.
Pictures (a) to (d) show meshes with a different number of elements. For each of these meshes the diodicities as well as the pressure differences were determined. The corresponding number of degree of freedoms are: (a) coarser mesh $DOF = 210\,028$; (b) coarse mesh $DOF = 473\,068$; (c) normal mesh $DOF = 1\,001\,156$; (d) fine mesh $DOF = 1\,318\,404$. The subfigures (e) and (f) show the resulting pressure differences for the forward and the backward directions. In subfigure (g), the resulting diodicities are shown. It becomes clear that the increase in the number of network elements does have an influence on the results. However, the results converge quite quickly. The mesh with the "normal" mesh size was used for all calculations. Further refinement does not bring any relevant improvement, but significantly increases the computational effort.

must be achieved. The results of the convergence study are presented in Fig. 3. It is clear that the mesh used for the simulations is sufficiently accurate, and further refinement does not provide noticeable further improvement.

1.2 Measurements

Additional measurement results on the valves which were not discussed in the main text, are presented afterwards.

1.2.1 Diodicities

In Fig. 3a of the paper the measured values of the diodicity are shown as a function of the Reynolds number for the different valve designs. The Reynolds number was calculated via:

$$Re = \frac{\varrho \cdot U \cdot d_{\mathrm{H}}}{\eta} \tag{1}$$

where ϱ is the density of the liquid, d_{H} is the hydraulic diameter, $U = \dot{V}/A_{\mathrm{in}}$ is the average velocity at the inlet, which is calculated as the ratio of the flow rate $\dot{V}$ and the area of the inlet A_{in} and η is the viscosity of the liquid. The hydraulic diameter is calculated via:

$$d_{\mathrm{H}} = \frac{2h_{\mathrm{C}}l_{\mathrm{C}}}{h_{\mathrm{C}} + l_{\mathrm{C}}} \tag{2}$$

where $h_{\mathrm{C}} = 130\,\mu\mathrm{m}$ denotes the etch depth of the valve and $l_{\mathrm{C}} = 1000\,\mu\mathrm{m}$ is the width of the inlet. Thus, the Reynolds number is calculated as usual for the characterization of Tesla valves, see e.g. [1]. However, we would like to point out that the flow velocity shows a strong spatial dependence and is strongly increased within the nozzle structure. Thus, a correct definition of the Reynolds number is difficult, as already discussed by Tao et al.[2].
Each measurement point in Fig. 3a of the main paper corresponds to the average of three individual measurements. The diodicity Di and the standard error SE of the diodicity are calculated as described by Dunlap et al.[3] using:

$$Di\left(\dot{V}\right) = \frac{\overline{\Delta p_{\mathrm{B}}}\left(\dot{V}\right)}{\overline{\Delta p_{\mathrm{F}}}\left(\dot{V}\right)} \tag{3}$$

$$\mathrm{SE}\left(Di\left(\dot{V}\right)\right) = \left|Di\left(\dot{V}\right)\right|\sqrt{\left(\frac{\sigma_{\mathrm{PB}}\left(\dot{V}\right)}{\overline{\Delta p_{\mathrm{B}}}\left(\dot{V}\right)}\right)^2 + \left(\frac{\sigma_{\mathrm{PF}}\left(\dot{V}\right)}{\overline{\Delta p_{\mathrm{F}}}\left(\dot{V}\right)}\right)^2} \tag{4}$$

where σ_{P} denotes the standard deviation and $\overline{\Delta p}$ is the mean value of the measured pressure values at the respective flow rate.

Parameter	Valve designs		
	V_{A}	V_{B}	V_{C}
SB	0.0002	0.0038	0.0012
MSV	0.0015	0.0015	0.0058
MSD	0.0017	0.0052	0.0070
r	0.953	0.944	0.988

Table 1 Parameters quantifying the deviation between the simulated and the measured data.

To more accurately quantify the error between the simulation and measured data, the squared bias SB, the mean squared variation MSV, the mean squared deviation MSD and the correlation coefficient r were calculated for the different valve designs. The calculation is based on the following equations [4]:

$$\begin{aligned}
SB &= \left(\frac{1}{N}\sum_{j=1}^{N}\left(Di_j^S - Di_j^M\right)\right)^2 = \left(\overline{Di^S} - \overline{Di^M}\right)^2 \\
MSV &= \frac{1}{N}\sum_{j=1}^{N}\left(\left(Di_j^S - \overline{Di^S}\right) - \left(Di_j^M - \overline{Di^M}\right)\right)^2 \\
MSD &= \frac{1}{N}\sum_{j=1}^{N}\left(Di_j^S - Di_j^M\right)^2 \\
r &= \frac{\frac{1}{N}\sum_{j=1}^{N}\left(Di_i^S - \overline{Di^S}\right)\left(Di_j^M - \overline{Di^M}\right)}{\sqrt{\frac{1}{N}\sum_{i=1}^{N}\left(Di_j^S - \overline{Di^S}\right)}\sqrt{\frac{1}{N}\sum_{j=1}^{N}\left(Di_j^M - \overline{Di^M}\right)}}
\end{aligned} \tag{5}$$

where Di^M denote the measured and Di^S the simulated values for the diodicities. The quantities with a bar denote the respective mean values. The squared bias SB indicates whether our simulation model can describe the measurement well. The smaller the value of MSV, the better the simulation can explain the deviations of the measurement from the mean. Both quantities add up to the mean squared deviation MSD. Thus, for a good agreement between measurement and simulation, the two quantities SB and MSV must be small at the same time. In addition, an r-value closer to 1 indicates a better agreement between the measurement and the simulation. The results for all the quantities are summarized in Table 1 for the different designs. They have also been mentioned in the caption of Fig. 3. The equations were added to the supplemental. It becomes clear that both SB and MSV in each case are low and that the r value is close to 1. Thus, we can speak of a good agreement between the measured and the simulated data. However, it also becomes clear that the deviations between the simulation and the measurements slightly increase with increasing diodicity.

1.2.2 Microscopic particle imaging velocimetry

Figures 4 and 5 compare simulated and the measured flow profiles for the valves V_B and V_C.

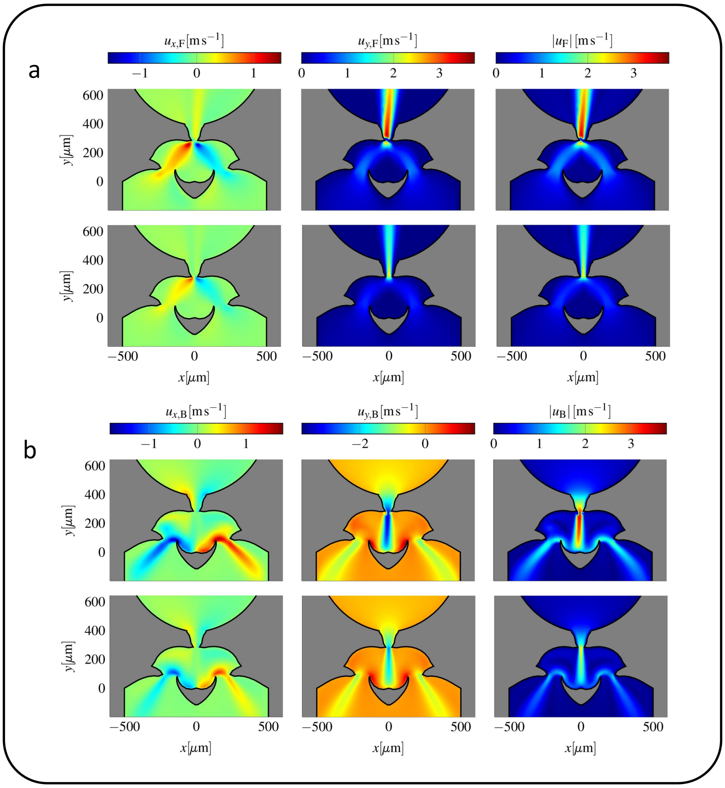

Fig. 4 Measured and simulated flow profiles for valve V_B
a and **b** Comparison between the flow profiles measured by μPIV and the simulated flow profiles for the valve V_B. In the upper rows of the two subfigures, the measured data are shown and in the bottom rows, the simulation results are presented. In all illustrations, the valve geometries were overlaid to facilitate visualization. The individual velocity components $u_{x,y}$ and the magnitude $|u|$ of the velocity vectors are displayed. Panels (a) and (b) show the flow profiles for the forward and the backward direction, respectively.

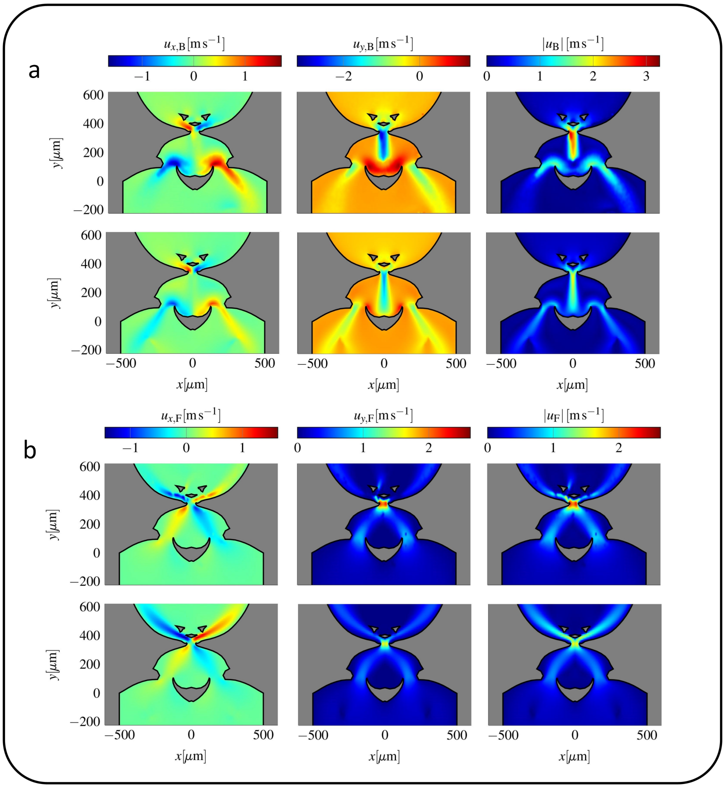

Fig. 5 Measured and simulated flow profiles for valve V_C
a and **b** Comparison between the flow profiles measured by µPIV and the simulated flow profiles for the valve V_C. See Fig. 4 for details.

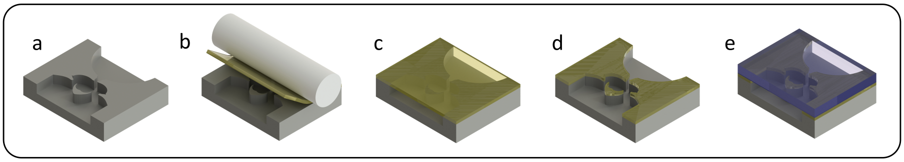

Fig. 6 Schematic representation of the lamination and bonding process. The silicon is shown in gray, the Ordyl in yellow and the glass in blue. (a) Structured silicon wafer. (b) A laminator is used to apply a thin layer ($d = 30\,\mu\text{m}$) of the Ordyl SY330 dry film resist to the structured wafer. (c) The resist is stiff enough to span the channels. (d) Using conventional lithography techniques, the Ordyl is exposed and subsequently wet-chemically developed. The resists remains only on the island structures as well as around the channel. (e) A glass wafer is then bonded to the Ordyl layer at a temperature of 150 °C and a force of 6 kN.

1.2.3 Fabrication

To further clarify the process of bonding using the Ordyl SY330, the process is illustrated in Fig. 6. It should also be mentioned that the the process described here enables also the bonding of two structured substrates. This is particularly relevant in the manufacturing of micropumps.

2 Supplemental Video

A video is available online showing the optimization process for valve V_B. At the beginning of each optimization stage, the used mesh is shown and then the calculated material distributions $\gamma_\mathrm{P} \leq 0.5$ are visualized after every 10th iteration. The upper half of each frame shows the flow profile in the backward direction and the lower half shows the flow profile for the forward direction. The same color scale is used for all frames of each optimization stage.

References

[1] Lin, S., Zhao, L., Guest, J. K., Weihs, T. P. & Liu, Z. Topology optimization of fixed-geometry fluid diodes. *Journal of Mechanical Design* **137** (8), 081402 (2015). https://doi.org/10.1115/1.4030297 .

[2] Tao, R., Ng, T., Su, Y. & Li, Z. A microfluidic rectifier for Newtonian fluids using asymmetric converging–diverging microchannels. *Physics of Fluids* **32** (5), 052010 (2020). https://doi.org/10.1063/5.0007200 .

[3] Dunlap, W. P. & Silver, N. C. Confidence intervals and standard errors for ratios of normal variables. *Behavior Research Methods, Instruments, & Computers* **18** (5), 469–471 (1986). https://doi.org/10.3758/BF03201412 .

[4] Kobayashi, K. & Salam, M. U. Comparing simulated and measured values using mean squared deviation and its components. *Agronomy Journal* **92** (2), 345–352 (2000). https://doi.org/https://doi.org/10.2134/agronj2000.922345x .
